# Predictors of Intention to Smoke among Junior High School Students in Shanghai, China: An Empirical Test of the Information-Motivation-Behavioral Skills (IMB) Model

**DOI:** 10.1371/journal.pone.0080482

**Published:** 2013-11-14

**Authors:** Chendi Zhu, Yong Cai, Jin Ma, Na Li, Jingfen Zhu, Yaping He, Pamela Redmon, Yun Qiao

**Affiliations:** 1 School of Public Health, Shanghai JiaoTong University, Shanghai, PR China; 2 Global Health Institute, Emory University, Atlanta, Georgia, United States of America; 3 Pudong Institute for Health Development, Shanghai, PR China; University of Sao Paulo, Brazil

## Abstract

**Background:**

Adolescent smoking is a worldwide problem that is particularly severe in low- and middle-income countries. Many endogenous and environmental factors affect the intention to smoke, so a comprehensive model is needed to understand the significance and relationship of predictors. The study aimed to test the associations among information-motivation-behavioral skills (IMB) model constructs as predictors of intention to smoke in junior high school students in Shanghai, China.

**Methods:**

We conducted a cross-sectional study of 16,500 junior high school students in Shanghai, China. Data on tobacco-related information, motivation, behavioral skills, and behaviors were collected from students. Structural equation model (SEM) was used to assess the IMB model.

**Results:**

The mean age of participants was 13.8 years old (standard deviation = 1.02; range 11–17). The experimental smoking rate among junior high school students was 6.6% and 8.7% of the participants expected that they would be smokers in 5 years. The IMB model provided acceptable fit to the data (comparative fit index = 0.984, root mean square error of approximation = 0.04). Intention to smoke was predicted by behavioral skills (β= 0.670, P < 0.001) and motivation (β= 0.095, P<0.001) among junior high school students.

**Conclusion:**

The IMB model provides a good understanding of the predictors of intention to smoke and it suggests future interventions among junior high school students should focus on improving motivation and behavioral skills.

## Introduction

Non-communicable diseases (NCDs) are the leading cause of mortality, contributing to 36 million of the 57 million global deaths in 2008 [1]. In China, tobacco use is one of the most preventable causes of NCD [2]. Tobacco causes about a million deaths per year in China, and the deaths are likely to increase to 3 million by the middle of this century [3,4]. Adolescent smoking has increased continuously in China, with the average age of onset of smoking has been decreasing [5,6]. Children and adolescents are the most vulnerable of experimental smokers (smoking one or two puffs of a cigarette) and are more likely to progress to current smokers [5]. Tobacco experimentation among school children aged 13-15 in Shanghai is nearly 7% and the estimated prevalence of current cigarette smoking is 28.1% among adults aged 15 years and above in China[7]. Once adolescents begin to experiment with smoking, they are likely to become regular smokers and the earlier individuals start smoking the higher risk of addiction in later life [8]. An understanding what lead adolescents decide or intent to smoke is an important issue on the future of tobacco control. 

Intention usually represents a decision to exert effort to perform the behavior [9]. Although there is a gap between intention and behavior, intention measurement remains a useful construct and is widely used in researches [10–12]. Intention to smoke is considered to be the major predictor of smoking behavior since numerous theories in social and health psychology assume that intention cause behaviors [11,12]. Cross-sectional and cohort studies have been used to evaluate the factors predicting adolescent smoking intention and behavior [7,8,10,13]. A previous study suggested that information, or one’s recognition of the health hazard of smoking, may indirectly affect smoking intention by changing their self-efficacy and reducing the positive expectancies [13]. Tobacco advertisements were associated with a significant increase in the probability of becoming a smoker [14]. In developing countries, adolescents are exposed to tobacco advertising, promotion through media, sports sponsorships everywhere and about 10% of young people have the experiences of being offered free cigarettes by the tobacco companies [15]. Other researchers have focused on adolescents’ perceived smoking environment, such as family smoking, friends’ smoking and the monitoring of smoking behavior by schools, which are critical predictors of smoking initiation [16-20]. In addition, adolescent normative expectations about cigarette smoking are important determinants of smoking initiation [21]. Adolescents with high expectations of the benefits of smoking are more likely to become smokers [22]. Collins found prior smoking experience was the most important predictor of future smoking confirmed that the best predictor of future behavior is past behavior [23]. In summary, intention to smoke among adolescents is influenced by personal factors and various external factors as well, including psychological, social and environmental factors. 

**Figure 1:**
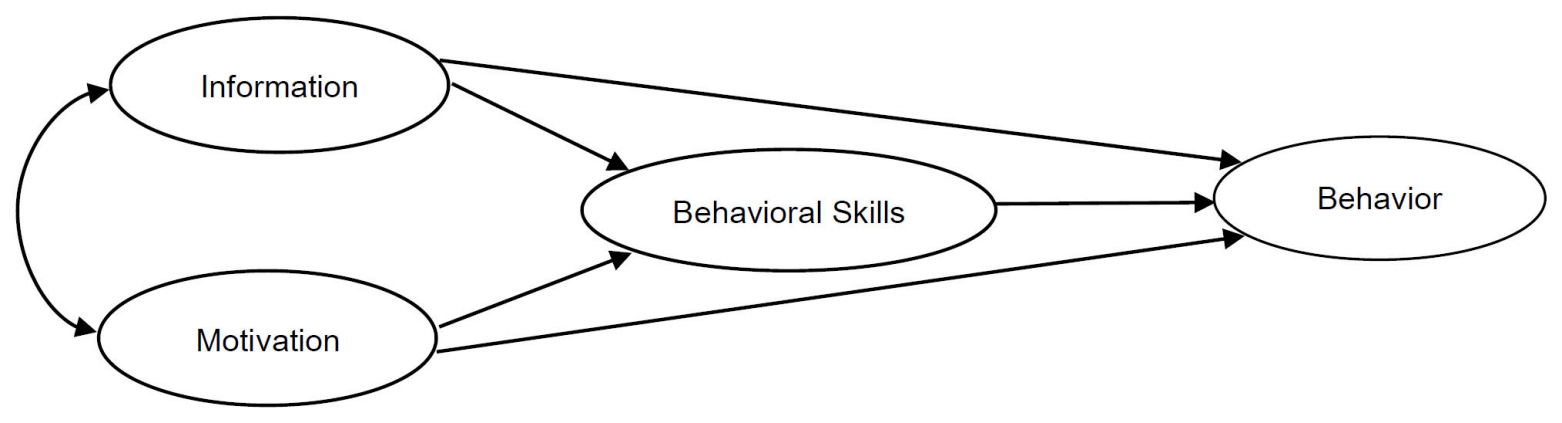
The IMB model.

With the recognition of these variables, several integrated models or theories have been applied to interpret the process of smoking behavior and intention. For example, the theory of planned behavior (TPB) and the Theory of Reasoned Action (TRA) have both been used to predict adolescent’s intention to smoke and smoking behavior [9]. The two theories both suggest that the proximal determinants of behavior are intentions to engage in the behavior [9,24]. Adolescents with a low perceived behavioral control (PBC) and perceiving a subjective norm approving of smoking will have a stronger intention to start smoking. Borrowed elements and theories from these earlier works, information-motivation-behavioral skills model (IMB model) has been constructed to explain complex health behaviors in the recent years [25,26]. This model was first proposed by Fisher to predict HIV prevention behaviors, and has been subsequently used to evaluate behaviors related to breast self-examination, female migrant workers’ reproductive health, and diabetes self-care [25–29]. Due to the complexity of the observed and latent multiple-factors related to smoking behavior, structural equation model (SEM) is often used in this field [12,30]. IMB model is also a SEM with four latent variables including information, motivation, behavioral skills, and behavior (Figure 1). Each latent variable is composed of one or more observed variables that can be evinced from questionnaire answers. IMB model is a causal model estimating the structural coefficients between constructs or latent variables [26,27]. To our knowledge, only one study has focused on intention to use smokeless tobacco by information-motivation-behavioral skills (IMB) model [13], and none have researched the intention to smoke among Chinese adolescents.

In this study, we used the Chinese version of the standard questionnaire, the Global Youth Tobacco Survey (GYTS), which identifies the key variables required by the IMB model. We assume that information, motivation (normative expectations, media exposure, and perceived smoking environment) will influence the participants’ skills (self-efficacy and previous smoking status), and predict intention to smoke (Figure 1). We also hypothesize that information and motivation may link directly with participants’ final behavior. The primary aims of this study are to identify the validity of IMB model and to test the associations among constructs as predictors of intention to smoke in junior high school students mostly aged 13-15 years.

## Methods

### Ethics

The Ethics Committee of School of Public Health, Shanghai Jiaotong University approved the study. Before enrollment in the study, all school organizers, students and their parents or guardians provided written informed consent, which included the objectives and procedures of the study, and potential risks and benefits of participation.

### Study Area

Shanghai, with a population of 23 million, is the largest city in China. A recent survey found that the current smoking rate in Shanghai residents was 23.5% in 2008 [31]. According to the data of the sixth population census from Shanghai Statistic Bureau in 2011, there are approximately 584,300 adolescents aged 13-15 years [7]. 

### Sample and procedure

A cross-sectional study of adolescents was conducted by asking students from junior high schools (usually aged 13-15years) in Shanghai to complete the Chinese version of GYTS. This questionnaire has been tested for its reliability and validity in preliminary research [7,32]. The GYTS is a school-based survey of specific geographic sites which can be countries, provinces, cities, or any other sampling frame [33]. GYTS uses a standardized methodology for sampling, participants selecting, field survey and data analysis to monitors teenage (usually aged 13-15years) smoking behavior [32].

Based on GYTS methodology, a two-stage cluster sample was conducted to produces representative samples in the current study. In the first stage, four districts of Shanghai were randomly selected and all junior high schools in these districts were included as the sampling frame. Sixteen schools were randomly chosen from these districts determined as the subjects of study. In the second sampling stage, 208 classes within chosen schools were randomly selected and all the students who attended school the day the survey was administered were eligible to participate. However, ninth-grade students were excluded from the samples, because they were preparing for the national senior high school entrance examination at the time of sampling. The sample size of the field survey was 17,132 students, from whom 16,500 usable questionnaires were collected for a response rate of 96.3%.

### Data collection

Trained researchers explained the study purpose and instructed students to fill the questionnaire. Participants were told that completing the questionnaire was voluntary and anonymous. All the students were given time to finish the questionnaire during regular school hours. 

### Measures

The key constructs of the IMB model included three latent variables: information, motivation, and behavioral skills. Intention to smoke was used as the main outcome behavior and dependent variable. Each latent variable of the IMB model was constructed with one or more observable variables that could be observed and measured directly.

### Information

Information was measured using four items with ‘yes’, ‘no’ or ‘do not know’ responses (e.g., ‘Do you think tobacco use will damage your health?’ ‘Do you think the second-hand smoke from others will damage your health?’). Negative answers of ‘no’ or responses of ‘do not know’ were scored as one, while positive answers of ‘yes’ were scored as two. Factor analysis showed equal coefficients for each of the four items, suggesting that the sum of these items could form a composite scale. The sum of each item’s score was converted into a total score as the scale of information (Cronbach’s alpha coefficient = 0.62; range of 4–8) where a higher score indicates the participants have access to more information.

### Motivation

The motivation was measured by three indexes. The first index, normative expectations about cigarette smoking, contained nine items involving interpersonal relationships, personality, charm, and image (e.g., ‘Do you think smokers are more attractive?’, ‘Do you think smokers are more elegant?’, ‘Do you think smokers are more popular?’). The answer to each question was assessed via a four-point Likert scale (1 = agree, 4 = disagree) and the sum of the scores for the nine items was taken as the index of normative expectations of tobacco use (EXPECT; Cronbach’s alpha coefficient = 0.73; range 9–36), where a higher score indicated a higher normative expectations about cigarette smoking. The second index, media exposure, contained nine items to assess exposure to tobacco-related advertisements and social activities among youth (e.g., ‘Have you seen tobacco-related advertisements on TV or at the movies during the past month?’, ‘Did you see a tobacco-related advertisement when you took part in a sports event or concert?’, ‘Have you seen a tobacco-related advertisement in a newspaper during the past month?’). The answer to each question was assessed via a four-point Likert scale (1 = never, 4 = always). The sum of the scores of these nine items was taken as an index of tobacco-related media exposure (MEDIA; Cronbach’s alpha coefficient = 0.68; range of 9–36), where a higher score indicated a higher level of media exposure to tobacco use. The third index was the perceived smoking environment which contained nine questions about the smoking behaviors of family members and friends, second-hand smoke exposure and school-based tobacco control (e.g., ‘Does your mother/father smoke cigarettes?’, ‘Does your best friend smoke cigarettes?’. ‘Do you suffer from second-hand smoke in the recent week?’, ‘Have you received school-based courses of health promotion on tobacco control?’). The answers were assessed by four-point Likert scales (1= never, 4=always). The sum of the scores of these nine items was taken as the index of perceived smoking environment. (ENVIRO; Cronbach’s alpha coefficient = 0.70; range of 9–36), where a higher score indicated a poorer tobacco control environment.

### Behavioral Skills

The behavioral skills were assessed on two scales. The first scale assessed self-efficacy to refuse tobacco from the participant’s peers by asking ‘Will you smoke if your best friend gives you cigarettes?’ and the response were measured on a four-point scale (1 = no, 2 = maybe not, 3 = maybe yes, 4 = yes). The scores indicated self-efficacy to refuse tobacco (EFFIC; range of 1–4) and indicated resistance to peer pressure smoking where a higher score indicates low self-efficacy. The second scale assessed prior experience with tobacco use including experimental smoking and current smoking. Collins found prior smoking experience was the most important predictor of future smoking [23]. The participants were asked two questions: ‘Have you ever tried or experimented with cigarette smoking, even one or two puffs?’, and ‘Have you used tobacco in the past 30 days’. A ‘no’ answer was scored as one and a ‘yes’ answer was scored as two. The sum of both scores indicated prior experience with tobacco (PRIOR; Cronbach’s alpha coefficient = 0.83; range of 2–4), where a higher score indicated more prior experience with tobacco use. 

### Intention to smoke

The final outcome indicated by the IMB model, intention to smoke, was assessed by two questions: ‘Will you smoke in the next 12 month?’, and ‘Will you smoke in the next 5 years?’ Answers were assessed by a 4-point Likert scale (1 = completely no, 4 = completely yes.) The sum of the scores of the two questions was taken as the index of intention to smoke (Cronbach’s alpha coefficient = 0.78; range of 2–8), where a higher score indicated the participants were more likely to report intention to smoke in the future.

## Statistical analysis

The ‘Complex Samples procedure’ of the Statistical Package for Social Sciences (SPSS vision 20.0) for Windows was used to perform the statistical analyses. A weighting factor was applied to each student record to adjust for non-responses (by school, class, and student) and variation in the probability of selection at the school and class levels [7]. The hypothetical IMB model was examined by the structural equation model (SEM) using the Amos 20.0 (IBM SPSS). The comparative fit index (CFI) and the root mean square error of approximation (RMSEA) were calculated. In theory, the CFI ranges from 0 to 1, where 1 indicates perfect fit, 0.9 indicates adequate fit, and 0.8 is considered marginal fit [34]. The RMSEA can range from 0 to ∞, where values ≤ 0.05 indicates close fit, ≤ 0.08 indicates a reasonable fit, and ≥ 0.1 indicates a poor fit [35,36]. RMSEA is sensitive to overfit, or may increase when too many associations have been set [37]. We also calculated the likelihood ratio by the chi-square test, but this test may be more affected by the sample size than CFI and RMSEA [38]. A preliminary confirmatory factor analysis (CFA) model was constructed to evaluate the factor structure and relationships among the IMB variables.

## Result

### Participant characteristics

A total 16,500 students completed the survey, with an average age of 13.8 years [95% confidence interval (CI): 13.7–13.9; SD = 1.02; range: 11–17 years]. As shown in Table 1, most of the students were 13–15 years old (61.9%; N = 10,061; 95% CI: 59.5–64.1). About half the participants were female (49.3%; N = 8,103, 95% CI: 48.4–50.1), 55.6% were from suburb areas (N = 9158; 95% CI: 53.1–58.0), 6.6% were experimental smokers (N = 1114; CI: 6.1–7.2), but only 1.0% were current smokers (N = 178; 95% CI: 0.8–1.2). Most students (95.9%; N = 15,812; 95% CI: 95.5–96.3) considered themselves as consistent nonsmokers over the next 12 months, but a slightly smaller number (91.3%; N = 15,037; 95% CI 90.6–91.9) responded they would not smoke over the next five years.

**Table 1:** Participant characteristics using the Complex Samples Procedure (N=16500）.

Characteristics	Number	Weighting% ( 95% CI*)
Gender		
Male	8397	50.7 (49.9–51.6)
Female	8103	49.3 (48.4–50.1)
Age(years)		
<13	4489	25.5 (22.8–28.4)
13-15	10061	61.9 (59.5–64.1)
>15	1950	12.6 (11.1–14.4)
Hometown		
Urban	7342	44.4 (42.0–46.9)
Suburb	9158	55.6 (53.1–58.0)
Experimental smoking		
Yes	1114	6.6 ( 6.1- 7.2)
No	15386	93.4 (92.8–93.9)
Current smoking		
Yes	178	1.0 (0.8- 1.2)
No	16322	99.0 (98.8–99.2)
Intention to smoke in the next 12 months		
No	15812	95.9 (95.5–96.3)
Maybe not	381	2.3 ( 2.0- 2.6)
Maybe Yes	240	1.4 ( 1.1- 1.6)
Yes	67	0.4 ( 0.3- 0.5)
Intention to smoke in the next 5 years		
No	15037	91.3 (90.6- 91.9)
Maybe not	720	4.3 (3.9- 4.6)
Maybe Yes	644	3.9 (3.5- 4.3)
Yes	99	0.6 (0.5- 0.7)

* CI: Confidence interval

### Validation of IMB model

**Figure 2:**
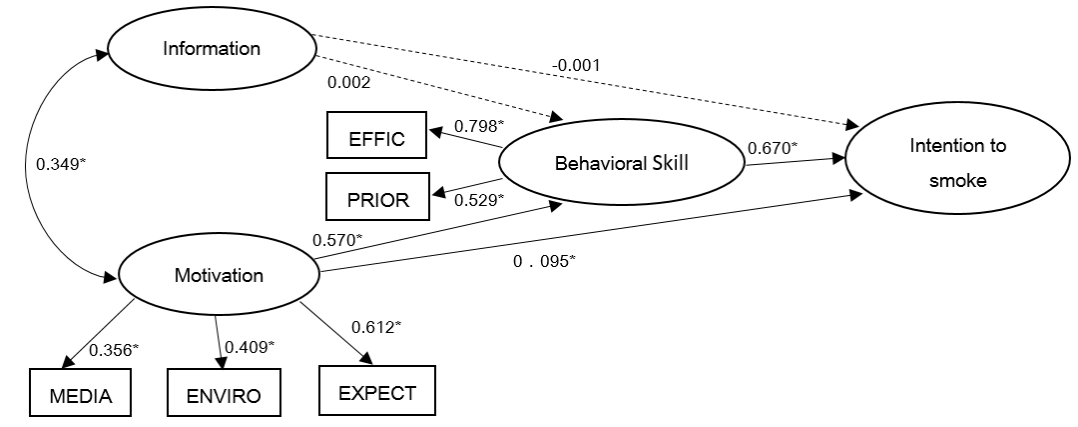
The preliminary IMB model predicting adolescent tobacco use among 16500 students. Oval represent multiple-indictor latent variables, rectangle represent single-indicator observable variables. Single-headed arrow means regression path. Double-headed arrow mean correlations. Regression coefficient are standardized (*means P<0.001). Dotted line indicates non-significant path from original IMB model.

The summary statistics for each part of the IMB model are shown in Table 2 and the predicting model of intention to smoke with parameters and paths significance is shown in Figure 2. The value of CFI was 0.984 indicated a good fit and the RMSEA was within an acceptable range of 0.05 or less (RMSEA = 0.04). χ^2^ = 271.0, df =10.

**Table 2:** Summary statistics of IMB model in confirmatory factor analyses using the Complex Samples Procedure (n=16500).

Scales	Mean (95% CI)	SD*
**Information**(Range 4–8)	6.24 (6.20–6.28)	1.70
**Motivation**		
Tobacco-related media exposure (MEDIA; Range:9–36)	14.75 (14.64–14.86)	3.30
Normative expectations about cigarette smoking (EXPECT;Range:9–36)	14.14 (14.01–14.27)	3.74
Perceived smoking environment (ENVIRO;Range:9–36)	15.81 (15.70–15.93)	3.94
**Behavioral Skills**		
Self-efficacy to refuse tobacco (EFFIC;Range:1–4)	3.09 (3.05–3.13)	1.51
Prior experience with tobacco use (PRIOR;Range:2–4)	2.08 (2.07–2.09)	0.31
**Intention to smoke** (Range: 2–8)	2.20 (2.18–2.22)	0.73

* SD: Standard deviation

The model indicated that behavioral skills (β = 0.670, P < 0.001) and motivation (β= 0.095, P < 0.001) were both predictors of intention to smoke. Furthermore, the model showed that motivation and behavioral skills were strongly related (β= 0.570, P < 0.001). Motivation was predicted by media exposure (β= 0.356, P < 0.001), perceived smoking environmental (β= 0.409, P < 0.001), and normative expectations (β= 0.612, P<0.001). Information was not significantly related to intention to smoke (β= -0.001, P>0.05) and behavioral skills (β=0.002, P>0.05). 

## Discussion

In the current study, the prevalence of smoking experimentation was 6.6% among junior high school students, which was consistent with the result of the previous study in Shanghai [7]. Other GYTS studies among students aged 13–15 years in different countries found experimental smoking rates ranged from 12.1% (Sri Lanka) to 73.6% (Ukraine) [39]. The wide variation could be attributed to the different cultural environment, religious norms, smoking control strategies, and availability of tobacco products in different countries. In this sample of junior high school students, the prevalence of current cigarette smoking is only 1% in Shanghai. However, nearly 9% of the participants admit to be the potential smokers in the coming 5 years，which suggests the urgency and necessity to conduct prevention programs among the target population. 

Understanding what leads the intention to smoke among junior high school students will contribute to future smoking prevention programs. The current study aims to utilize the well-constructed IMB model to interpret intention to smoke among these students. It indicates that intention to smoke can be well explained by the IMB model. Students with more smoking motivation and less tobacco refusal skills are more likely to report intention to smoke in the future. Tobacco related information is indirectly associated with intention to smoke, but was significantly correlated with motivation. These results are consistent with those of other studies and suggest that information is an important but unnecessary precursor to health behavior [27,28]. A meta-analysis of adolescent smoking prevention programs also found that providing information was capable of changing one’s knowledge, but had a limited effect on attitudes and behavior [40]. Information is not directly associated with behavioral skills, which suggests that telling adolescents that smoking is hazardous does not provide the skills to decrease the intention to smoke. Traditional intervention which only focused on providing information that smoking is a health hazard from the media or other people might be insufficient [40]. In addition, the IMB model confirms that perceived smoking environment such as parents and friends smoking, media tobacco advertising, and normative expectations all affect the motivation to use tobacco among adolescents. This is consistent with prior review that four single prevention programs (school, media, community and environmental) all showed effectiveness in influencing adolescent intention in some aspects [41]. The model indicates that behavioral skills and motivation directly contribute to intention to smoke. Thus, media advocacy, school courses, community programs should all be included in an effective smoking prevention program, in addition to refusal skills training. 

Given that the IMB model can predict intention to smoke well, it may be used as a basis for the design of integrated adolescent smoking prevention in the future. We propose several sessions that should include behaviorally relevant information about smoking hazards, normative expectations from several motivation-related respects, and most importantly, exercises targeting the development of refusal skills in different situations [42].

There are several limitations in this study. First, the study was conducted among adolescents living in a large city and the results might not be generalized to individuals coming from smaller rural areas. Second, all ninth-grade students were excluded from the survey, which might limit generalization of the research finding. Third, Students might misreport their behaviors or attitudes in the self-reporting data, however, reliability and validity studies have indicated good test-retest and good fit of the results for the questionnaire we used [7,32]. Finally, the results of this study were based on cross-sectional data which limited utility for causality analysis. We tested IMB model by using structural equation model with specified paths and measured the constructs from cross-sectional data, which could strengthen the associations between constructs. However, longitudinal data is needed to observe the effects of information, motivation and behavioral skills on changes in the intention to smoke in the future research.

Despite these limitations, our study is, to the best of our knowledge, the first time the predictive utility of the IMB model has been evaluated for intention to smoke in China. This model indicates which crucial variables are related to intention to smoke, and how the variables are associated with it. Importantly, the IMB model shows that smoking motivation and behavioral skills are directly associated with smoking intention. Together, these findings have implications for the development of more integrated adolescent tobacco prevention programs in the future.
